# Chronic inflammation-associated genomic instability paves the way for human esophageal carcinogenesis

**DOI:** 10.18632/oncotarget.8356

**Published:** 2016-03-25

**Authors:** Runhua Lin, Chong Zhang, Jiaxuan Zheng, Dongping Tian, Zhijin Lei, Donglin Chen, Zexin Xu, Min Su

**Affiliations:** ^1^ Institute of Clinical Pathology, Guangdong Provincial Key Laboratory of Infectious Disease and Molecular Immunopathology, Shantou University Medical College, Shantou, Guangdong, 515031, PR China

**Keywords:** esophageal carcinogenesis, chronic inflammation, oxidative DNA damage, DNA double-strand breaks, genomic instability

## Abstract

Chronic inflammation is associated with increased risk of cancer development, whereas the link between chronic inflammation and esophageal carcinogenesis is still obscure heretofore. This study aimed to investigate the relationship between chronic inflammation and DNA damage, as well as the possible role of DNA damage in esophageal carcinogenic process. Endoscopic esophageal biopsies from 109 individuals from Chaoshan littoral, a high-risk region for esophageal squamous cell carcinoma (ESCC), were examined to evaluate the association between chronic inflammation and histological severity, while additional 204 esophageal non-tumor samples from patients with ESCC were collected. Immunohistochemistry was performed to detect the oxidative DNA damage and DNA double-strand breaks (DSBs). Significantly positive correlation was observed between degree of chronic inflammation and esophageal precursor lesions (*r_s_* = 0.37, *P* < 0.01). Immunohistochemical analysis showed that oxidative DNA damage level was positively correlated with the degree of chronic inflammation (*r_s_* = 0.21, *P* < 0.05). Moreover, the level of oxidative DNA damage positively correlated with histological severity (*r_s_* = 0.49, *P* < 0.01). We found that the extent of DSBs was progressively increased with inflammation degree (*P* < 0.01) and the progression of precancerous lesions (*P* < 0.001). Collectively, these findings provide evidence linking chronic inflammation-associated genomic instability with esophageal carcinogenesis and suggest possibilities for early detection and intervention of esophageal carcinogenesis.

## INTRODUCTION

Esophageal cancer exhibits a common malignancy with a poor prognosis, ranking the 10th most common cancers and taking a toll of ~400,000 deaths in 2012 worldwide [1], and esophageal squamous cell carcinoma (ESCC), accounting for ~90% esophageal cancer, is the predominant histological subtype throughout the world [2]. The hugest burden of ESCC occurs in the “Asian esophageal cancer belt”, which extends from Caspian littoral of Iran, east to China, and north to Russia [3]. Our previous epidemiological study has shown an extremely high incidence of ESCC (74.47/100,000) in Nan'ao Island of Shantou, an isolated Chaoshan littoral region of southern China, from 1995 to 2004 [4], highlighting an urgent need for deeper understanding of its pathogenesis. In most cases, the development of ESCC is associated with esophageal squamous dysplasia. As a consequence, esophageal squamous dysplasia is regarded as the precursor lesion and risk factor for ESCC. A 13-year prospective follow-up study has demonstrated that patients with mild, moderate, or severe dysplasia have a risk of ESCC that is increased by a factor of 2.9, 9.8, or 28.3, respectively [5]. Hence, it is of great importance to explore the underlying mechanisms for the development of esophageal dysplasia.

The association between chronic inflammation and tumorigenesis was first proposed by Rudolf Virchow over a century ago, when he noticed the presence of inflammatory cells in neoplastic tissues [6]. Since then, accumulating evidence from preclinical and clinical studies has shown that recurrent and persistent chronic inflammation may be a causative factor for oncogenesis [7]. It has previously been estimated that chronic infection and related inflammation contributed to approximately 20% to 25% of all cancer cases worldwide [8].

Chronic inflammation-induced reactive oxygen and nitrogen species (RONS) could cause damage to important cellular components (e.g., DNA, proteins and lipids), which consequently contributes to malignant cell transformation [9]. According to our previous study regarding chronic inflammation-related DNA damage response (DDR) in gastric cardia carcinogenesis [10], the level of DDR was increased with the progression of precancerous lesions in human gastric cardia tissues.

All the aforementioned evidence begs the question of how chronic inflammation influences tumor initiation. In the study presented herein, we therefore investigated the relationship between DNA damage status and degree of chronic inflammation as well as precursor lesions in human esophageal tissues. Our findings demonstrated that the level of DNA damage was positively correlated with degree of chronic inflammation and precursor lesions. Taken together, chronic inflammation-related DNA damage (genomic instability) may trigger the initiation of esophageal squamous dysplasia.

## RESULTS

### Chronic inflammation correlated with esophageal precancerous lesions

**Figure 1:**
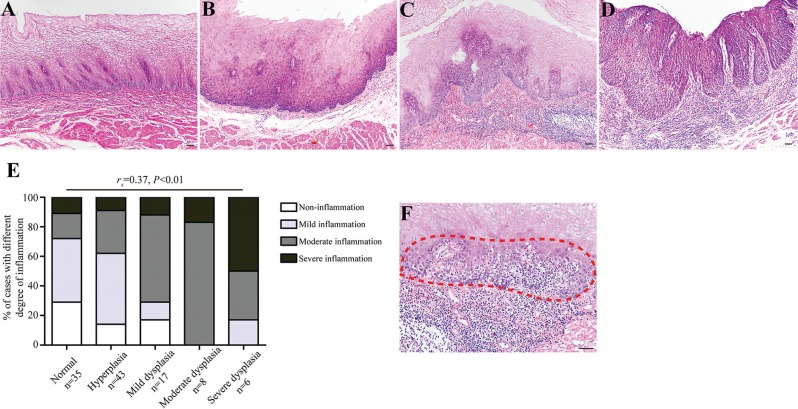
The degree of chronic inflammation correlates with esophageal histological severity (**A**–**D**) Representative images showing normal epithelium without evident inflammation (Panel A), hyperplastic epithelium with mild inflammation (Panel B), mild dysplastic epithelium with severe inflammation (Panel C), and severe dysplastic epithelium with severe inflammation (Panel D), respectively. (**E**) Esophageal precursor lesions were associated with the degree of chronic inflammation. For statistical analysis, Spearman rank correlation analysis was used, with *P* values indicated in the figure. (**F**) The association between chronic inflammation and esophageal histological severity was evidenced by microscopic appearance of intraepithelial inflammation, intraepithelial infiltrating inflammatory cells and dysplastic esophageal epithelial cells are indicated with red dashed line. Scale bars correspond to 50 μm in A–D and F.

To clarify the role of chronic inflammation during the course of esophageal carcinogenesis, we examined a total of 109 endoscopic esophageal biopsies from non-tumor individuals (Table 1). We noticed that esophageal precancerous lesions (e.g., mild, moderate, and severe dysplasia) were frequently accompanied by varying degree of chronic inflammation. The above histological evidence aroused our attention on the association between chronic inflammation and esophageal tumorigenesis. As expected, severe chronic inflammation was commonly observed in severe dysplastic esophageal epithelia (Figure 1A–1D). Correlation analysis revealed that chronic inflammation was positively correlated with histological severity (*r*_s_ = 0.37, *P* < 0.01) (Figure 1E). Interestingly, we found that inflammatory cells migrated into esophageal squamous epithelia, and the neighboring epithelial cells constantly exhibited dysplastic changes (Figure 1F). One possible explanation could be that chronic inflammation may serve as a critical role in malignant transformation of esophageal epithelial cells instead of an accidental phenomenon.

**Table 1:** Clinicopathological data of 109 endoscopic biopsy specimens

Variable	Value
Age, yrs, *n* = 109	54.2 ± 16.2
Sex, *n* = 109	
Male (%)	71 (65)
Female (%)	38 (35)
Histological condition, *n* = 109	
Normal (%)	35 (32)
Hyperplasia (%)	43 (39)
Mild dysplasia (%)	17 (16)
Moderate dysplasia (%)	8 (7)
Severe dysplasia (%)	6 (6)
Degree of inflammation, *n* = 109	
Normal (%)	19 (17)
Mild (%)	38 (34)
Moderate (%)	36 (34)
Severe (%)	16 (15)

### Oxidative DNA damage correlated with chronic inflammation and histological severity

**Figure 2:**
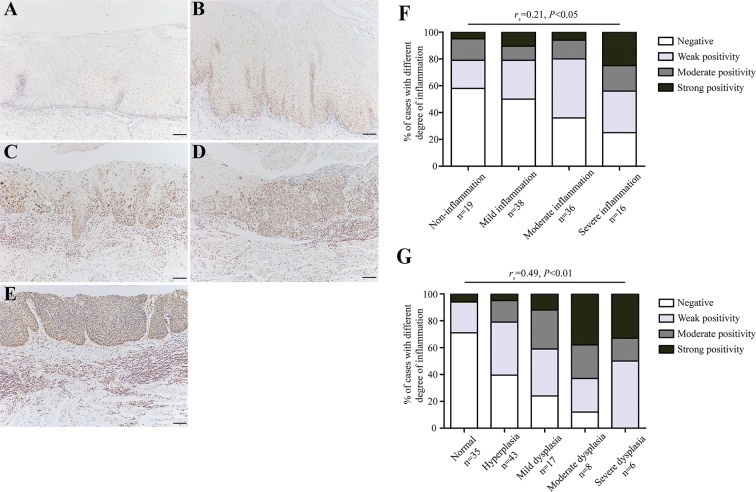
The level of oxidative DNA damage correlates with the degree of chronic inflammation as well as esophageal histological severity (**A**–**E**) Representative images showing 8-OHdG immunostaining in tissues with non-inflammtion (normal tissue) (Panel A), mild inflammation (hyperplasia) (Panel B), moderate inflammation (mild dysplasia) (Panel C), moderate inflammation (severe dysplasia) (Panel D), and severe inflammation (severe dysplasia) (Panel E), respectively. (**F**–**G**) Correlation analysis for the level of oxidative DNA damage and the degree of chronic inflammation (F), as well as esophageal histological severity (G). For statistical analysis, Spearman rank correlation analysis was used, with *P* values indicated in the figure. Scale bars correspond to 50 μm in A–E.

Although inflammation is a critical function of the innate immune system, chronic inflammation is often accompanied by the excessive formation of reactive oxygen species (ROS) that may potentially inflict damage on nucleic acids, proteins and lipids in neighboring healthy epithelial and stromal cells [11]. As a result, this process can lead to mutations in tumor-related genes, which increases cancer risk. We, therefore, speculate that ROS-induced oxidative DNA damage may explain the close relationship between chronic inflammation and esophageal precancerous lesions. We then stained all the 109 cases of endoscopic esophageal biopsies using anti-8-OHdG antibody for detection of oxidative DNA damage. Intriguingly, the immunostaining intensity of 8-OHdG was prominently stronger in tissues with higher degree of chronic inflammation, whereas only a small fraction of non-inflammation tissues showed positive immunostaining (Figure 2A–2E). Correlation analysis of the immunohistochemical results demonstrated that the immunostaining intensity of 8-OHdG was positively correlated with the degree of chronic inflammation (*r*_s_ = 0.21, *P* = 0.03) (Figure 2F), indicating that higher level of oxidative DNA damage occurred during chronic inflammatory process, particularly in the setting of severe chronic inflammation.

Based on aforementioned results, we have reasons to hypothesize that oxidative DNA damage ought to be evident in esophageal preneoplastic lesions in case of its involvement in tumor initiation. To clarify this, we evaluated the oxidative DNA damage status in esophageal epithelial tissues grouped by different pathological changes. Being consistent with our assumption, the level of oxidative DNA damage increased progressively in the sequential stages from histologically normal esophageal epithelia to dysplastic esophageal epithelia (Figure 2A–2E). In addition, correlation analysis demonstrated a significantly positive association between oxidative DNA damage and esophageal precancerous lesions (*r*_s_ = 0.49, *P* < 0.01) (Figure 2G). These results suggest that chronic inflammation-related oxidative DNA damage probably triggers the initiation of esophageal carcinogenesis.

### The level of DSBs increased with esophageal histological severity

**Figure 3:**
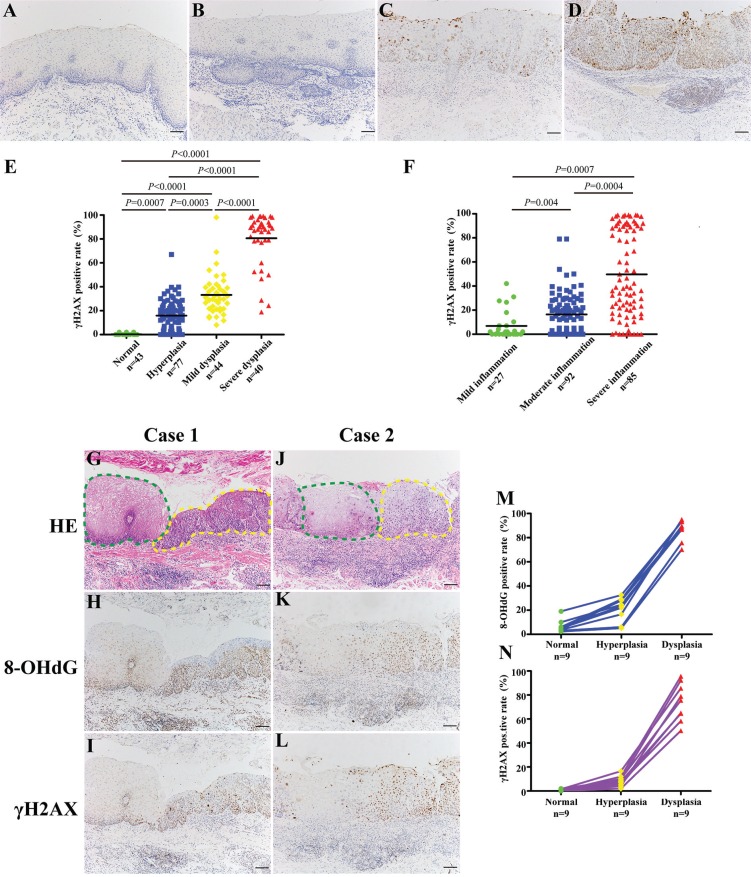
The level of DSBs correlates with esophageal histological severity (**A**–**D**) Representative images of γH2AX immunostaining in normal epithelium (Panel A), hyperplastic epithelium (Panel B), mild dysplastic epithelium (Panel C), and severe dysplastic epithelium (Panel D), respectively. (**E**–**F**) Quantification of γH2AX positive cells in tissue samples with different histological severity (E) and different degree of chronic inflammation (F). For statistical analysis, one-way ANOVA was used, with *P* values indicated in the figure. Tamhane's T2 tests were selected to identify specific differences between each two groups. For multiple comparisons, the *P* values of or less than 0.008 (E) or 0.016 (F) were considered statistically significant adjusted by Bonferroni correction. Black bars indicate mean values. (G–L) Representative images showing immunostaining of 8-OHdG and γH2AX in samples with transitional pathological changes from the same ESCC patient (**Case 1** and **Case 2**). Green dashed line indicates normal epithelium; yellow dashed line indicates dysplastic epithelium. (M, N) Quantification of 8-OHdG (M) and γH2AX (N) positive cells in samples with transitional pathological changes from the same patient. Scale bars correspond to 50 μm in A–D, G–L.

We have confirmed that the accumulation of oxidative DNA damage in inflamed or dysplastic esophageal tissues. Additionally, we have previously reported that chronic inflammation-related DNA damage response is significantly increased in dysplastic tissues of human gastric cardia compared with that in normal gastric cardia tissues [10]. The phosphorylation of serine 139 in the C-terminal tail of the histone H2A variant, γH2AX, is a rapid and sensitive cellular response to the presence of DSBs [12], and its aggregation, in a way, is a predictor of genomic instability [13]. Hence, we attempted to evaluate the status of DSBs in esophageal tissues with different histological severity during esophageal carcinogenic process (Table 2). Importantly, immunostaining of γH2AX was evident in dysplastic epithelial cells, especially in severe dysplastic epithelial cells, whereas the immunoreactivity was almost absent in histologically normal tissues (Figure 3A–3D). Immunohistochemical analysis also confirmed this phenomenon with statistical significance (*P* < 0.001) (Figure 3E). Similarly, immunohistochemical analysis of γH2AX in tissues with different inflammation status demonstrated that the positive rates of γH2AX were significantly higher in samples affected by severe inflammation than that in tissues with milder inflammation (*P* < 0.01) (Figure 3F).

**Table 2:** Clinicopathological data of 204 tumor-surrounding non-malignant samples

Variable	Value
Age, yrs, *n* = 204	59.7 ± 9.1
Sex, *n* = 204	
Male (%)	168 (82)
Female (%)	36 (18)
Histological condition, *n* = 204	
Normal (%)	43 (21)
Hyperplasia (%)	77 (38)
Mild dysplasia (%)	44 (21)
Severe dysplasia (%)	40 (20)
Degree of inflammation, *n* = 204	
Normal (%)	0 (0)
Mild (%)	27 (13)
Moderate (%)	92 (45)
Severe (%)	85 (42)

### DNA damage in samples with different histological severity from the same ESCC patient

To rule out the potential influence of genetic background, we selected samples with transitional pathological changes from 9 ESCC patients to explore the DNA damage status during esophageal carcinogenic process. Not surprisingly, both the level of oxidative DNA damage and DSBs increased with esophageal histological severity (Figure 3G–3L). Immunohistochemical analysis revealed that immunostaining of 8-OHdG and γH2AX reached significantly higher positive rates than that in normal tissues (*P* < 0.05) (Figure 3M–3N). These data provide strong pathological evidence that DNA damage may play critical role in the initiation of esophageal malignant transformation.

## DISCUSSION

The present work was designed to evaluate the association between chronic inflammation-related DNA damage and esophageal premalignant lesions. In this study, we observed significantly positive correlation between chronic inflammation and esophageal precursor lesions. Subsequently, immunohistochemical analyses demonstrated that the levels of oxidative DNA damage and DSBs, evident as positive immunostaining of 8-OHdG and γH2AX, respectively, paralleled with esophageal histological severity. These findings suggest that chronic inflammation-related genomic instability may trigger the malignant transformation of esophageal epithelial cells.

Chronic inflammation has long been considered as an important risk factor for many human cancers [14–17]. Our present study shows a close association between chronic inflammation and esophageal precancerous lesions, providing histological basis for our further investigation on the link between chronic inflammation and esophageal carcinogenesis.

Histopathological features of chronic inflammation are predominance of macrophages and lymphocytes. A prolonged inflammatory microenvironment provides a constant supply of ROS, leading to the formation of oxidative DNA damage. According to previous investigations, the level of oxidative DNA damage is found increased in the inflamed gastric mucosae of *Helicobacter pylori*-infected people and in the inflamed colonic mucosae of rats [18, 19]. Similarly, our results also reveal that the level of oxidative DNA damage in esophageal epithelial cells was positively correlated with degree of chronic inflammation, providing a rationale for the hypothesis that persistent existence of chronic inflammation could trigger oxidative DNA damage on the surrounding healthy epithelial cells.

Carcinogenesis is a long and multistep process including initiation (selection of a mutated cell), promotion (selective expansion of the initiated cell), and progression as a consequence of an imbalance between cell proliferation and cell death [20]. Oxidative damage to DNA by ROS is frequently postulated to give rise to mutations in tumor-related genes involved in essential cellular processes including proliferation, cell cycle checkpoints, apoptosis, and DNA repair. Our data show that oxidative DNA damage was positively correlated with esophageal precursor lesions, implying that the accumulation of oxidative DNA damage in esophageal epithelial cells, provided lacking of timely and accurate repair, may ultimately contribute to esophageal tumorigenesis.

**Figure 4:**
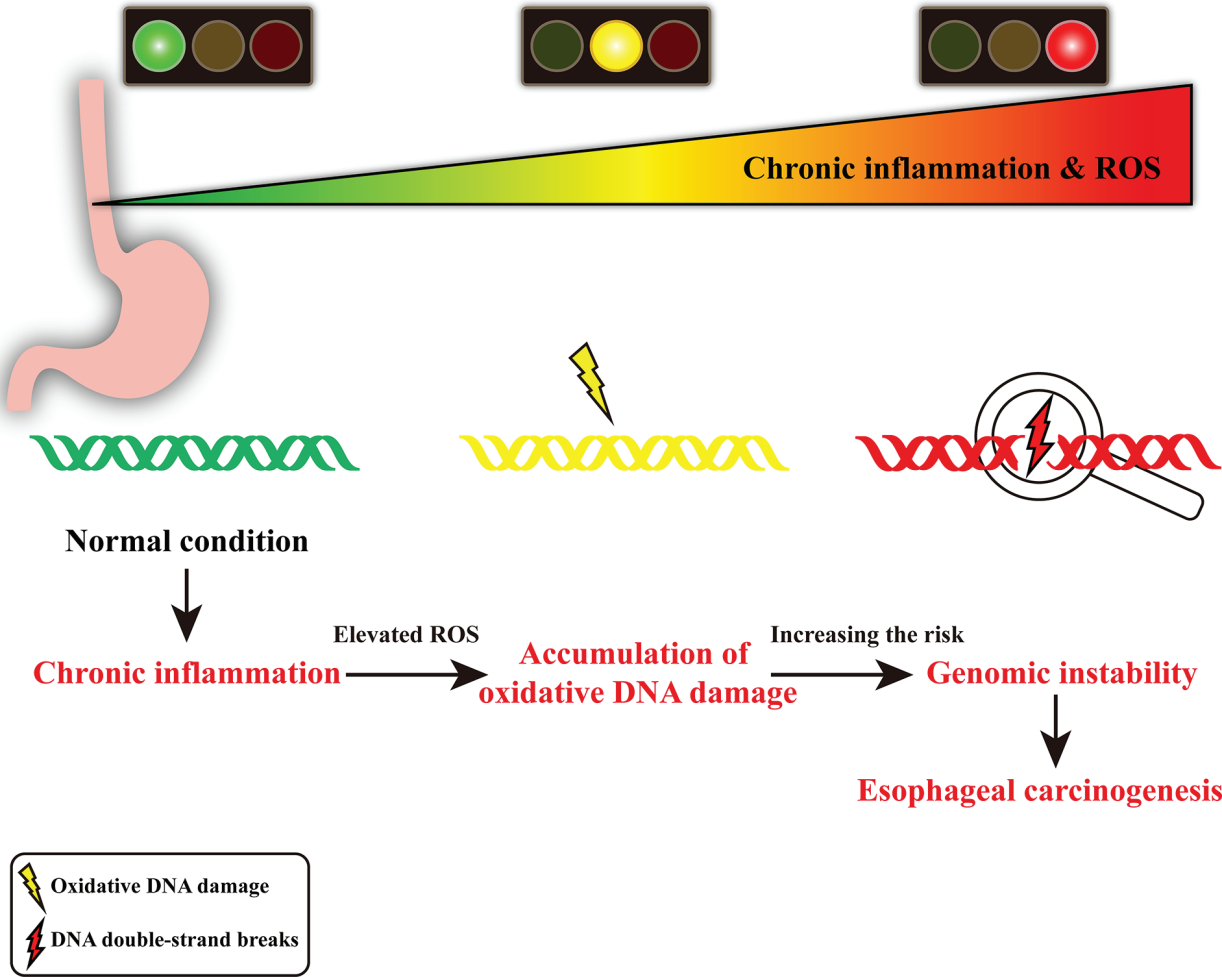
Schematic diagram of chronic inflammation-related DNA damage gears up esophageal carcinogenesis In chronic inflammatory microenvironment, the infiltrating inflammatory cells may produce DNA-damaging product (such as ROS), resulting in oxidative DNA damage and DNA double-strand breaks. Elevated level of DNA double-strand breaks may lead to genomic instability, contributing to esophageal carcinogenesis.

Genomic instability is a common characteristic of human cancers [21, 22] and plays a critical role in tumor predisposition. Of the many classes of DNA damage, DNA double-strand breaks (DSBs) is a serious lesion that can cause genomic instability, which may lead to cancer development [23]. In our present study, it is noteworthy to point out that the level of DSBs is significantly higher in dysplastic esophageal tissues compared with that in normal esophageal tissues. Furthermore, we also observe this phenomenon in samples with transitional pathological changes from the same ESCC patient. Thus, we speculate that chronic inflammation-associated genomic instability, evident as accumulation of γH2AX foci, may contribute to esophageal carcinogenesis (Figure 4). Collectively, these results provide evidence linking chronic inflammation-associated genomic instability with esophageal carcinogenesis and suggest possibilities for early detection and intervention of esophageal carcinogenesis.

## MATERIALS AND METHODS

### Esophageal mucosal samples collected from non-tumor individuals

A total of 109 endoscopic biopsy specimens of esophagus from tumor-free individuals from Chaoshan littoral were collected for examination (i.e. relationship between chronic inflammation and precursor lesions, immunostaining for 8-OHdG).

### Esophageal mucosal samples collected from ESCC patients

A total of 204 esophageal tissue samples from ESCC patients with no prior radio- or chemotherapy were collected from Cancer Hospital of Shantou University Medical College from January 2008 to December 2014. For each patient, tumor-surrounding non-malignant tissues (within 2 cm from tumor) and distant non-malignant tissues (more than 5 cm away from tumor) were analyzed for DSBs status.

Additionally, we also obtained samples involving sequential pathological changes (normal-hyperplasia-dysplasia) from the same patient (9 ESCC patients) to evaluate the DNA damage status in the process of esophageal carcinogenesis.

This study was approved by Ethics Committee of Shantou University Medical College, and informed consents were obtained from patients or their families.

### Grading of esophageal epithelial dysplasia

All samples were fixed, dehydrated and paraffin embedded, and presence of any epithelial changes deviating from normal esophageal epithelium were verified by two pathologists with hematoxylin and eosin staining. *Hyperplasia* indicated that an increase in the number of esophageal epithelial cells resulting in increased layers of the epithelium. *Dysplasia* was defined as nuclear atypia (enlargement, hyperchromasia, and pleomorphism), loss of normal cell polarity, but without invasion through the basement membrane (*mild dysplasia*: dysplastic cells within one-third layers of the epithelium; *moderate dysplasia*: dysplastic cells covering over one-third layers of epithelium, but confined to two-thirds layers of epithelium; *severe dysplasia*: dysplastic cells covering over two-thirds layers of epithelium).

### Inflammation analysis

The chronic inflammation degree of esophageal epithelium was categorized into four groups according to the density and affected areas of chronic inflammatory cells (lymphocyte and macrophage) as follows: *none* denoted that less than 10 inflammtory cells per high power field confined to the lamina propria; *mild* refered to that less than 100 inflammatory cells per high power field only confined to the lamina propria and with no infiltrating into the epithelial layer; *moderate* signified that less than 300 inflammatory cells per high power field located in the lamina propria, and less than 20 inflammatory cells located in esophageal epithelium; *severe* was judged by that lymphoid follicles appeared in lamina propria with more than 300 inflammatory cells per high power field and leukocytes diffusely infiltrated into the epithelial layer with more than 20 inflammatory cells per high power field.

### Immunohistochemistry

Immunohistochemistry was performed to confirm the presence of oxidative DNA damage and DNA damage response. Briefly, formalin-fixed, paraffin-embedded (FFPE) tissue blocks were sectioned at 4-μm thickness and then used to perform immunohistochemistry. Tissue sections were deparaffinized in xylene, then rehydrated through graded ethanol to water, and immersed in phosphate buffered saline (PBS). Subsequently, antigen retrieval was performed in a pressure cooker for 2 minutes at 125°C and endogenous peroxidase activity was quenched by 3% H_2_O_2_ in distilled water for 15 min. After three washes in PBS, sections were incubated overnight with anti-8-OHdG (mouse, dilution 1:300, Santa Cruz) and anti-γH2AX (mouse, dilution 1:600, Cell Signaling Technology) at 4°C in a humidified chamber followed by incubation for 30 min with goat anti-mouse/rabbit at 37°C. After washing, peroxidase substrate (DAB) was added, slides were washed in water and counterstained with hematoxylin. Slides were then washed, dehydrated and mounted using cover slips.

Images were captured using a Leica IM50 microscope at ×400, and five different fields for each index (8-OHdG and γH2AX) were selected in each sample. Due to the nuclear and cytoplasmic immunoreactivity of 8-OHdG, immunoreactivity was scored based on the percentage of positive epithelial cells (0: < 10%; 1: 11%–25%; 2: 26%–50%; 3: 51%–75%; 4: 76%–100%). The intensity was scored as negative (0), weak (1), moderate (2) and strong (3). The corresponding labeling index was computed as product of percentage of positive cells multiplied by immnostainin intensity with highest score of 12. The final evaluation of 8-OHdG expression was classified as four grades: score 0 for negative; score 1–4 for weak positivity; score 5–8 for moderate positivity; score 9–12 for strong positivity. For the quantification of γH2AX, the number of positive nuclei and total nuclei in images was counted and the corresponding immunostaining positive rates were computed as positive nuclei/total nuclei ×100%.

### Statistical analysis

The statistical analyses were performed using SPSS 13.0 software package (SPSS, Chicago, IL). Spearman rank correlation analysis was used to assess the association between chronic inflammation level and esophageal histological severity, chronic inflammation level and oxidative DNA damage, oxidative DNA damage and esophageal histological severity. A two-tailed *P* value of or less than 0.05 was considered statistically significant. One-way analysis of variance (ANOVA) was applied for γH2AX positive rates among the multiple groups. LSD (least significance difference) and Tamhane's T2 tests were selected to identify specific differences between each two groups for equal and unequal variance data, respectively. For multiple comparisons, *P* values were adjusted by Bonferroni correction.
